# Recent advances in the identification of related factors and preventive strategies of hip fracture

**DOI:** 10.3389/fpubh.2023.1006527

**Published:** 2023-03-13

**Authors:** Yaohui Yu, Yudan Wang, Xiaoli Hou, Faming Tian

**Affiliations:** School of Public Health, North China University of Science and Technology, Tangshan, Hebei, China

**Keywords:** osteoporosis, hip fracture, risk factors, protective factors, prevention measures

## Abstract

Hip fracture is the most devastating type of osteoporosis-related fracture, and is a major worldwide public health problem with a high socioeconomic burden, morbidity rate, and mortality rate. Thus, it is crucial to uncover the risk factors and protective factors to create a hip fracture prevention strategy. In addition to a briefly review of some well accepted risk and protective factors of hip fracture, this review mainly summarized the recent advances in the identification of emerging risk or protective factors for hip fracture, in terms of regional differences in medical services, diseases, drugs, mechanical load, neuromuscular mass, genes, blood types, cultural differences. This review provides a comprehensive review of the associated factors and effective prevention measures for hip fracture, and discusses issues that need further investigation. These issues include the determination of the influencing mechanism of risk factors triggering hip fracture and their interlinked correlation with other factors, as well as the confirmation or correction of emerging factors associated with hip fracture, particularly those that are still controversial. These recent findings will aid in optimizing the strategy for preventing hip fracture.

## 1. Introduction

Hip fracture is a common injury among older adults, which are associated with potential loss of autonomy, long-term disability and increased risk of mortality, resulting in both significant personal and socioeconomic burden (1, 2). Therefore, comprehensive screening of risk factors of hip fracture and optimization of fracture risk prediction system are of great significance for early prevention of hip fracture.

Although low BMD is an accept sign to identify people with an elevated risk, it is not reasonable to screen out all people at imminent, intermediate, and long-term risk for hip fracture by measurement of BMD alone, because BMD measurement does not capture microstructural deterioration which increases fragility disproportionate to the bone loss, leading to the mild BMD defects in postmenopausal osteopenia or so-called “normal” BMD women (3). Therefore, BMD cannot completely help us predict the occurrence of fragility fracture.

At present, related studies have shown that the relationship between the risk factors of hip fracture is also complex, and is related to the causes of the risk factors of hip fracture. In addition to some genetical and immutable conditions like age, gender, ethnicity and family history of fracture, a series of modifiable unhealth behaviors, as well some diseases, particularly those affect bone metabolism and their treatment can also lead to risk factors for hip fracture. For example, the natural deterioration of tissue structure and the decline in physiological function of various systems in the elderly are associated with an increased risk of hip fractures (4). In addition, several chronic health conditions and long-term use of a variety of medications are associated with both fall and bone health (5).

Based on numerous epidemiological data, this review focuses on the latest progress in identifying factors related to hip fracture and evaluating the correlations between hip fracture and other factors, which will help to modify behavior and identify high-risk groups, thereby developing a sound public health strategy to reduce the incidence of hip fracture.

## 2. Methods

### 2.1. Literature search

Five databases were searched by computer, including PubMed, Web of Science, Cochrane library database, Embase. At the same time, experts in the field of orthopeadic surgery were consulted to supplement and obtain relevant literature. The retrieval time limit is from the establishment of the database to December 31, 2021. Search terms were predefined to allow a comprehensive search strategy that included text fields within records, and the search strategy was based on a combination of “hip,” “fracture,” “cataclasis,” “bone,” “hip,” “protective factor,” “risk factor,” “influencing factor,” etc. We used Boolean operators (within each axis, we combined keywords with the “OR” operator to expand the search, and we then linked the search strategies for the two axes with the “AND” operator to narrow the search). For the first three databases, the retrieval strategy is shown in Appendix.

### 2.2. Inclusion and exclusion criteria

Inclusion criteria: (1) Types of studies: original cross-sectional studies, randomized controlled studies and case-control studies. (2) Types of participants: population with hip fracture. (3) The outcome of influencing factors: divided into the risk factors and protective ones.

**Figure 1:**
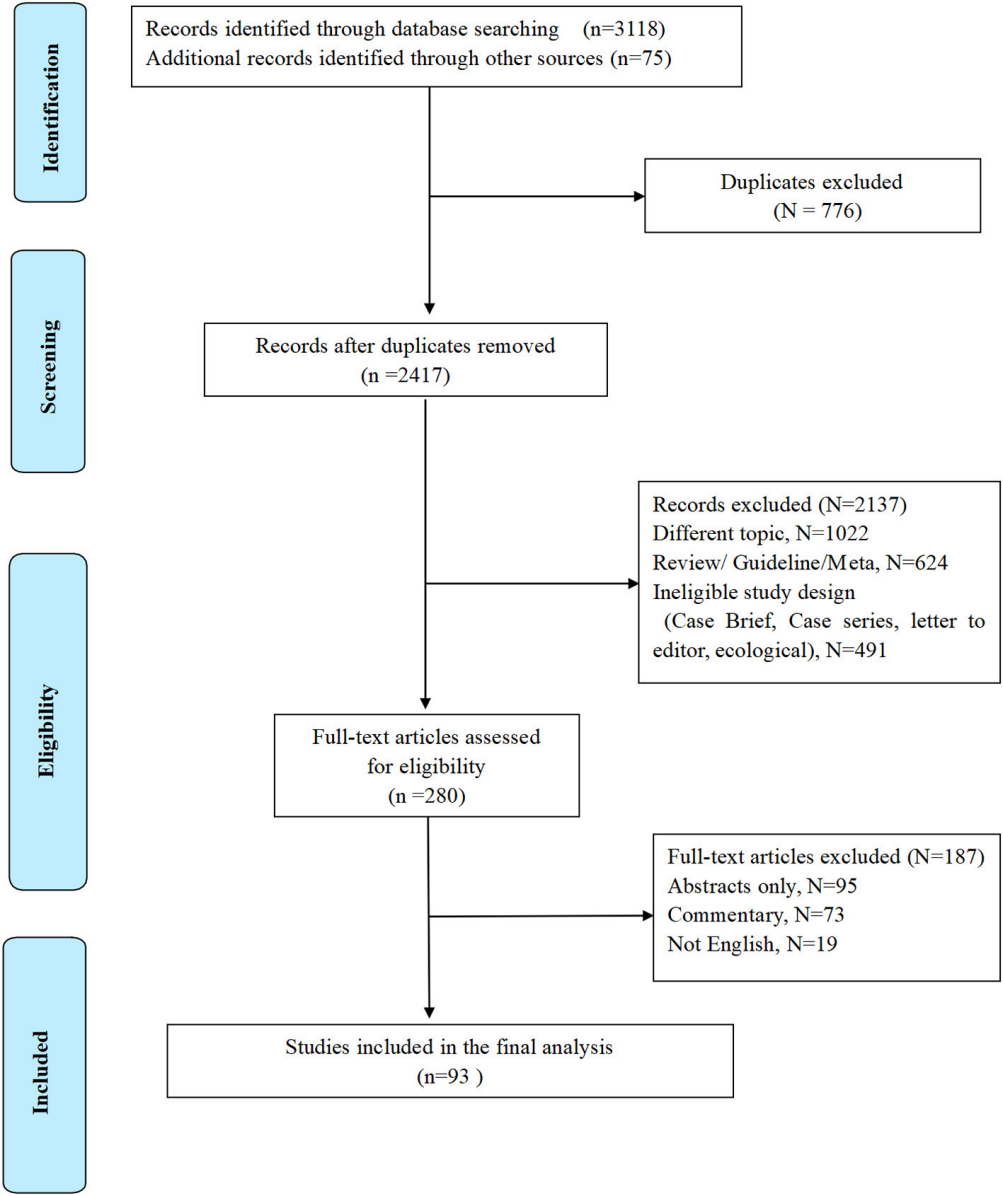
Flow diagram of study selection.

Literature exclusion criteria: (1) Case report, review, systematic evaluation, and meta-analysis. (2) Repeated published and poor-quality literature. (3) The information is incomplete, and the relevant data cannot be obtained or missing (Figure 1). PICOS framework is presented in Table 1.

**Table 1:** PICOS framework of the study for the identification of related factors of hip fracture.

**Parameter**	**Description**
Population	Healthy population, population with hip fracture
Intervention/ exposure	A series of risk or protective factors of hip fracture
Comparison	Patients in hip fracture and healthy control population
Outcome	The occurrence of hip fracture
Study design	Cross-sectional studies, randomized controlled studies and case-control studies

### 2.3. Data extraction and quality assessment

First, the title information of relevant literature was retrieved through the retrieval strategy, and Endnote X9 software was used for literature management. For the qualified literature finally selected, yaohui Yu and yudan Wang independently extracted the research information and then comprehensively evaluated the included references, thereafter independently evaluated the risk of bias included in the study and cross-checked the results. Xiaoli Hou decide the final conclusion by discussion (xiaoli Hou) when Yu and Wang got different opinions. The quality of the included articles were assessed by the Joanna Briggs Institute (JBI) quality assessment tool (6). The JBI offers a set of critical appraisal checklists for different study designs. The main focus of the appraisal checklist is on methodological rigor, avoidance of bias including selection bias, and information bias, appropriate use of statistics, and appropriate reporting, etc. Additionally, the risk of bias of the included studies was assessed using the ‘risk of bias assessment tool for non-randomized studies' (RoBANS) (7). The RoBANS tool focused on six major domains to address selection bias, performance bias, detection bias, attrition bias, and reporting bias (Table 2).

**Table 2:** Characteristics of included representative articles, summary of the main findings, and their quality assessment.

**References**	**Region; country**	**Study design; sample size**	**Study population (gender and age)**	**Study period (year)**	**Main findings related to factors causing hip fracture**	**Quality assessment scores**
Mayhew et al. (8)	Canada	Cross-sectional study	77 total population Age: Female 68.5 (41.0–75.0) Age: Male 70.0 (44.0–76.5)	1.00	Mechanical load and bone geometric parameters (femoral neck cortical stability)	9
Dargent-Molina et al. (9)	France	Cohort study	7,575 total population Female 77.8 (78.8–79.8)	1.94	Neuromuscular and visual impairments	8
Puttnam et al. (10)	America	Cohort study	22,180 total population Age: [mean (SD) 70.4 (6.7)]	8.00	Antihypertensive Medications	10
Kiel et al. (11)	America	Cohort study	1,042 total population Age: [mean (SD) 85.0 (7.0)]	1.60	Hip protector	9
Follis et al. (12)	America	Cohort study	11,020 postmenopausal women Age: [mean (SD) 62 (7.0)]	8.00	Psychosocial stress	10

## 3. Results

### 3.1. Well-accepted risk factors

**Figure 2:**
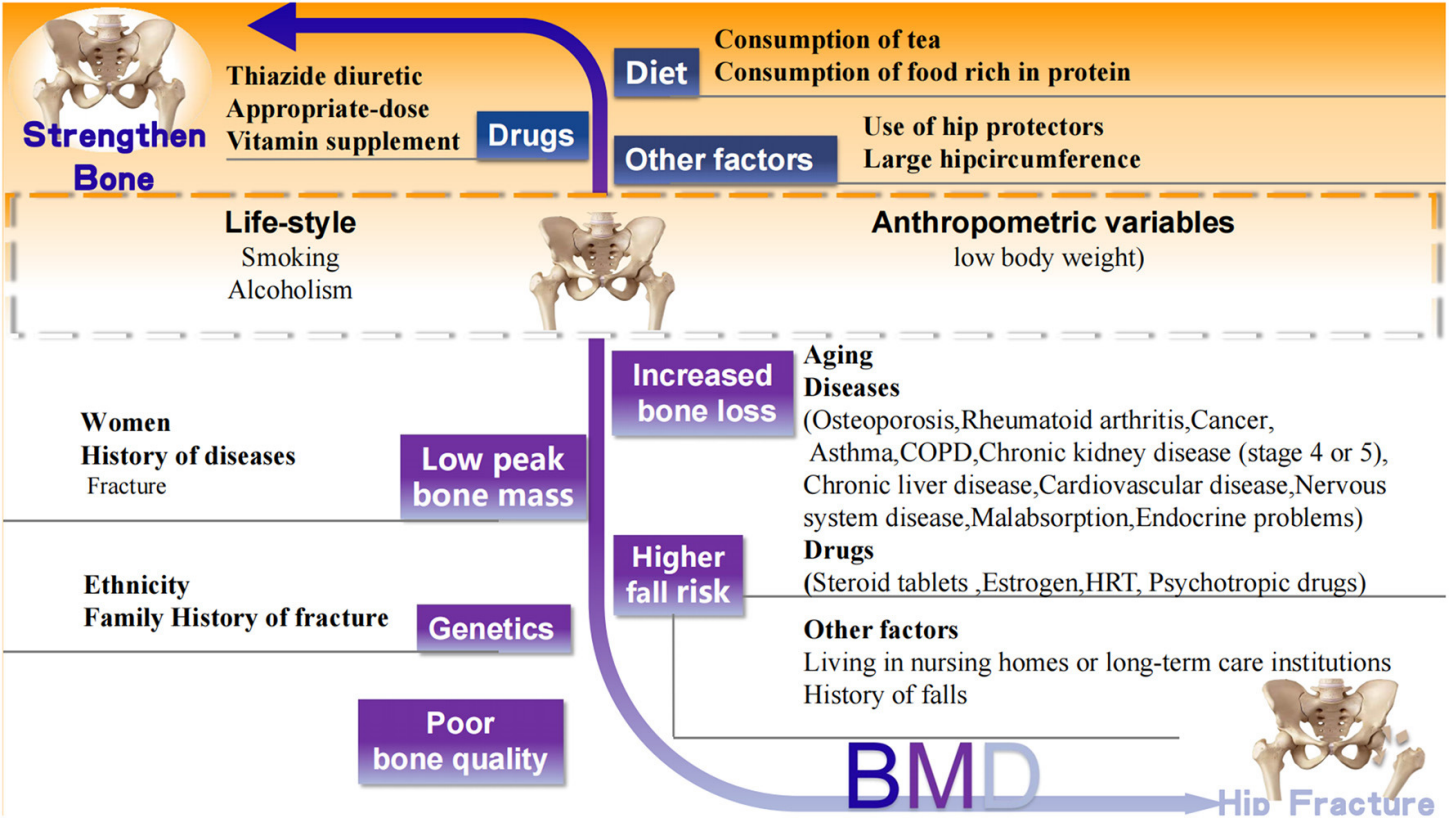
Well-accepted factors associated with hip fracture. Black words: well accepted risk (white background) and protective (orange background) factors related to hip fracture. CODP, chronic obstructive pulmonary disease; HRT, Hormone replacement therapy.

The well-accepted risk factors for hip fracture are summarized based on the World Health Organization (WHO) fracture risk assessment tool FRAX (www.shef.ac.uk/FRAX) and the other two osteoporotic fracture risk assessment scales Garvan Fracture Risk Calculator (www.qfracture.org) and QFracture (www.qfracture.org). Risk factors positively correlated with hip fracture include age, women, Caucasian ethnicity, smoking, alcoholism, low body weight, living in nursing homes or long-term care institutions, past fracture, family history of fracture, osteoporosis, rheumatoid arthritis, cancer, asthma, chronic obstructive pulmonary disease (COPD), chronic liver disease, chronic kidney disease (stage 4 or 5), cardiovascular disease [(heart attack, angina, stroke or transient ischemic attack (TIA)], nervous system disease (epilepsy, dementia, and Parkinson's disease), malabsorption (Crohn's disease, ulcerative colitis, coeliac disease, steatorrhea, or blind loop syndrome), endocrine problems (diabetes, thyrotoxicosis, hyperparathyroidism, and Cushing's syndrome), use of steroid tablets regularly, use of estrogen only or Hormone replacement therapy (HRT), use of psychotropic drugs such as benzodiazepines and barbiturates and history of falls (Figure 2).

### 3.2. Recently confirmed risk factors

**Figure 3:**
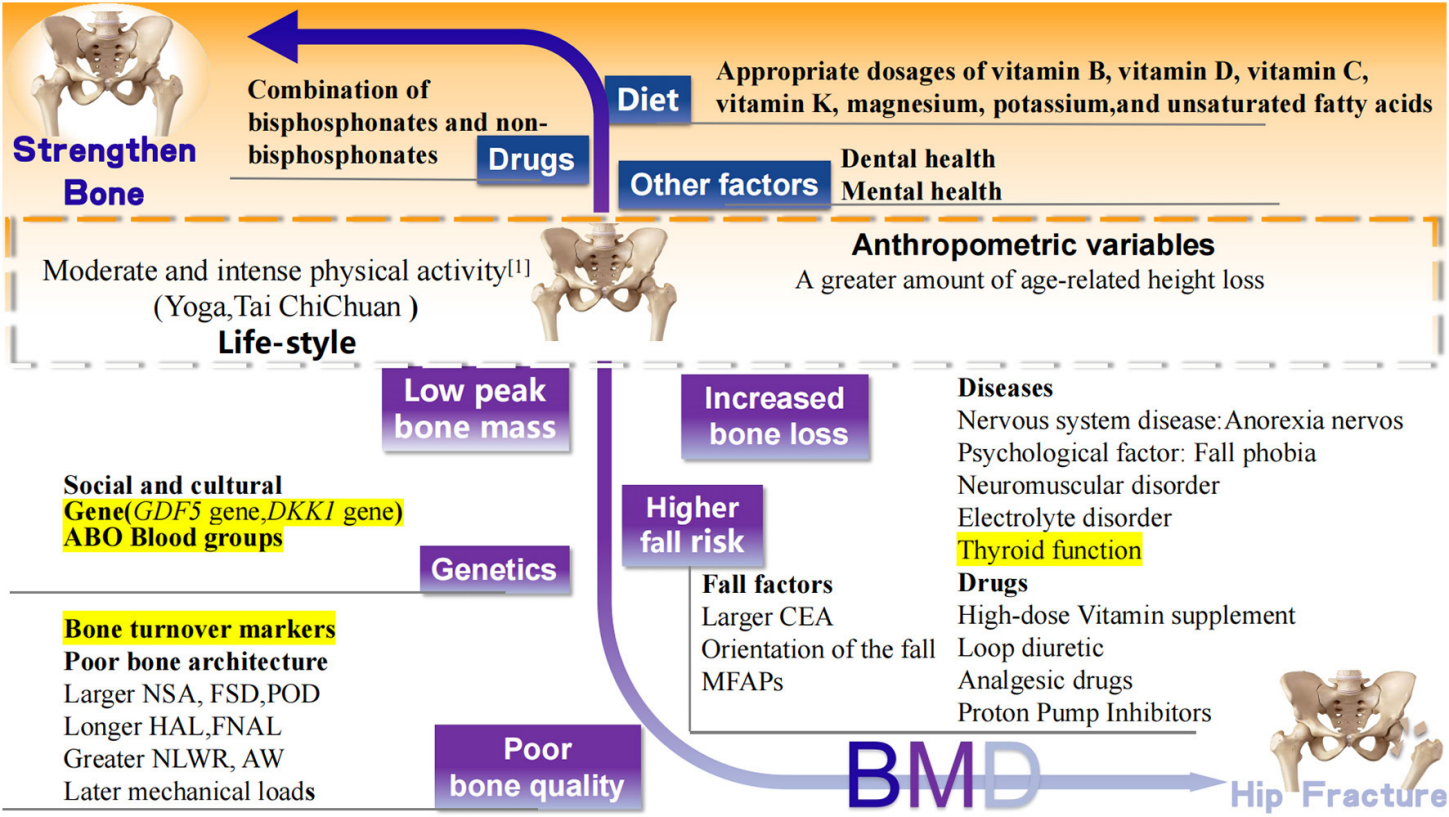
Recently confirmed factors associated with hip fracture. Black words: well accepted risk (white background), protective (orange background) factors, and controversial (yellow background) factors related to hip fracture. BMD, bone mineral density; MFAPs, meteorological factors and air pollutants; GDF5, Growth differentiation factor 5; DKK1, Dickkopf 1 gene; CEA, central edge angle; NSK, neck shaft angle; FSD, femoral shaft diameter; POR, pelvic outer diameter; HAL, length of hip axis; FNAL, femoral neck axial length; NLWR, neck length-width ratio; AW, acetabular width.

In addition to those above-mentioned well-accepted risk factors of hip fracture, recent studies have reported newly confirmed risk factors, including regional differences in medical services, some new diseases, drugs, and other variables that affect bone strength (Figure 3).

#### 3.2.1. Regional differences in medical services

It is reported that the process of urbanization may be parallel to the increasing hip fracture rate in most areas. A nationwide data analysis in Norway showed that the highest tertile of urbanization degree (city), compared to the lowest (rural), was related to a 23 and 24% increase in hip fracture risk in men and women, respectively (13). In a systematic review included 14 cohort studies and 1 case-control study, identified moderate evidence for rural residents have lower risk of hip fracture compared to urban residents (14, 15).

The reasons for such a phenomenon remain poorly understood, but may include decreased physical activity, soil sealing, calcium and vitamin D deficiencies, less sun exposure, and changes in BMD (16–18).

However, on the contrast, a series of studies support a beneficial effect of urbanizationon bone. Rural to urban migration as an adult is associated with higher BMD and greater predicted hip strength (19).

Compared with rural areas, the social level in urban areas is higher. It is reported that low socio-economic status has been confirmed as a risk factor for hip fracture (20). The results of a Chinese study show that income level is positively correlated with awareness of the perceived risk of osteoporosis (21). This suggests that people with higher incomes are more likely to be aware of hip fractures and maintain a healthy lifestyle and diet, which will be beneficial to the prevention of hip fractures. Further, urbanization is most often equal to higher levels of healthcare services. A recent study reported that regional differences in healthcare services are closely related to the risk of hip fracture, which may be due to the relative improvement of healthcare service in urban areas with a prosperous social economy (22). In this context, narrowing the gap of healthcare services level between rural and urban areas should be encouraged to reduce the fracture risk in rural areas.

Therefore, process of urbanization is a double-edged sword for the occurrence of hip fracture. It is important to recognize the factors influencing hip fracture and improve the awareness of preventive strategies to enhance the strengths and avoid the weaknesses of urbanization.

#### 3.2.2. Diseases

It is well established that BMD and other risk factors can be used to predict fracture risk (23). Recent research has found that diseases and drugs are also closely related to changes in BMD and the risk of hip fracture.

##### 3.2.2.1. Anorexia nervosa

A recent study found that anorexia nervosa (AN) is closely related to low BMD and an increased risk of hip fracture (24). Studies have shown that the bone loss rate of the hip joint of amenorrhea women with anorexia nervosa is 2.4% per year (25, 26). AN is a mental illness, long-term malnutrition is accompanied by severe bone loss, followed by hypogonadism and growth hormone resistance, which becomes an important intermediary factor for bone loss in patients with AN (27, 28). The lack of soft tissue around the trochanter of the femur due to insufficient calorie intake and the inability of individuals to maintain normal weight increases the risk of hip fracture in patients with AN (29). In addition, 50–60% of women with the disease do not recover until more than 20 years after initial diagnosis (30). In view of the long-term nature of the disease and the lack of approved treatment for bone loss in this population, future studies are necessary to optimize treatment strategies for osteopenia and increased risk of fracture in anorexia nervosa.

##### 3.2.2.2. Electrolyte disorders

With the advent of the “aging world”, an increasing number of studies have confirmed that electrolyte disorders affect the occurrence of fracture. Among these electrolyte disorders, hypokalemia is most frequently discussed. Hypokalemia may damage bone health by affecting bone turnover and low BMD, thus increasing the risk of fracture (31). An experimental study found that the decrease of bone trabecula and cortical bone led to abnormal bone histomorphology. This study found that hyponatremia could stimulate osteoclast formation and increase absorptive activity. This may be due to the increased level of circulating arginine-vasopressin hormone released from the posterior pituitary. The chronic increase of this hormone can also stimulate osteoclast activity and lead to bone resorption (32). A meta-analysis showed that hyponatremia is significantly correlated with fracture, osteoporosis, and higher mortality (33). Furthermore, patients with hyponatremia are more likely to fall than the general population, and the incidence of hyponatremia is increased in the older adult population, which strengthens the connection between hyponatremia and fracture (34). Chronic hyponatremia is also a risk factor for hip fracture in patients with chronic kidney disease who are older than 60 years (35). However, the risk of hip fracture in patients with chronic kidney disease is closely related to high levels of thyroid hormones (36). This suggests that doctors should be aware of the risk of hyponatremia. Further studies are needed to assess the risk of osteoporosis or fracture after hyponatremia treatment.

Although the mechanism of the effect of the above-mentioned diseases on hip fracture is complex and difficult to control, it helps to identify people at high risk of hip fracture to promote the prevention of hip fracture in patients with AN, hyponatremia, and hyponatremia.

#### 3.2.3. Drugs

A popular current research topic is the effect of drugs on the individual bone mass and the risk of hip fracture.

##### 3.2.3.1. Antihypertensive drugs

A large randomized clinical trial provided evidence of a beneficial effect of thiazide-type diuretic therapy in reducing hip and pelvic fracture risk compared with treatment with other antihypertensive medications (9). The latest study found that the use of thiazides (hydrochlorothiazide, bendroflumethiazide, or fixed drug combinations containing a thiazide) is effective in reducing the risk of hip fracture, while treatment with loop diuretics can increase the risk of fracture (37). Long-term administration of medullary loop diuretics leads to urinary calcium loss and/or PTH-driven bone turnover. Ultimately leading to a decrease in hip BMD in older men (38, 39). In addition, angiotensin-converting enzyme inhibitors, angiotensin receptor blockers, and beta-adrenergic blockers are risk factors for falls in the older adult population (40). There is evidence that different classes of medications might contribute to falls in older adults *via* orthostatic hypotension. Diuretics are associated with hypovolemia and orthostatic hypotension (41–43), which is a risk factor for both falls and syncope (44, 45). Clinicians should carefully consider the risk of falls in their selection of drugs for hypertension and the clinical use of hypertensive drugs. This should be monitored in older adults initiating antihypertensive therapy particularly among those with a prior history or other risk factors of falls to prevent the occurrence of hip fracture.

##### 3.2.3.2. Analgesic drugs

Hip fracture causes a massive amount of pain that is controlled with opioids in most cases (46). However, recent studies have found that opioids may increase the incidence of secondary hip fracture; a meta-analysis confirmed this finding and suggested that this may be related to the cognitive impairment, lethargy, and sedation caused by opioids (47). Thus, the use of opioids increases the likelihood of falls and leads to fracture (48). Among patients on hemodialysis in the United States, opioid use increases the risk of hip fracture regardless of the duration of intake (49). Weak opioids also affect the occurrence of hip fracture. Tramadol is an analgesic that is a weak opioid receptor. Although the risks of tramadol addiction and dependence are very small compared with commonly used weak opioid drugs (such as codeine) or commonly used non-steroidal anti-inflammatory drugs (such as naproxen, ibuprofen, celecoxib, and etoricoxib), tramadol is more likely to cause hip fracture (50). Overall, opioids and other analgesics should be used cautiously to control the pain associated with hip fracture to avoid the occurrence of secondary hip fracture.

##### 3.2.3.3. Vitamin supplements

Vitamin supplements are very popular among the general population, but dose control is important. Patients who receive high doses of vitamin B6 (≥35 mg/d) and vitamin B12 (≥20 μg/d) have a higher risk of hip fracture than those who receive appropriate doses of these vitamins (51). Similarly, the biological explanation for the effect on bone strength caused by higher and lower intake of nicotinic acid (a B3 vitamin) is unclear. High doses of vitamin B6 supplementation can cause neuromuscular damage and increase the risk of falls. Moreover, vitamin B6 may invalidate the regulatory effect of estrogen on steroid receptors, accelerate bone loss, and even cause damage (52). Vitamin D and calcium play important roles in bone development and are essential and necessary for bone health. Low levels of serum 25(OH)D may be associated with bone loss, lower bone mineral density (BMD) and higher bone turnover (53). A study containing 400 hip fractures found a dose-related increase in hip fracture risk for lower serum 25 (OH) D levels [OR = 1.33 (95%CI, 1.06–1.68) for each 25 nmol/l decrease] (54), while another study concluded that there was a significantly reduced risk of hip fracture in those with 25(OH)D levels ≥62.5 nmol/L compared to levels below this [RR = 0.64 (95%CI, 0.48–0.89)] (55). However, high doses bolus vitamin D supplements of 100,000 IU (2.5 mg) monthly over 2.5–4.2 years do not prevent falls and hip fracture, but rather increase the risk of falls (56–58). Therefore, vitamin intake and supplement should be based on their own needs and scientific advice to prevent the occurrence of the hip fracture, a recent meta-analysis suggested daily vitamin D dose of 800–1,000 IU was the most probable way to reduce the fracture and fall risk (59).

##### 3.2.3.4. Proton pump inhibitors

Proton pump inhibitors (PPIs) are widely used in several acid-related gastrointestinal disorders. *In vivo* studies have suggested that gastric suppression by PPIs could result in decreased intestinal calcium absorption. Subsequently, there have been concerns that the chronic use of a PPIs is associated with an increased risk of fracture, Previous studies on hip fracture risk associated with the use of PPIs have been inconsistent (60, 61). However, recent studies have agreed that the use of PPIs increases the risk of hip fractures. The impairment of bone health has been shown in both Caucasian (62) and Asian cohorts (63, 64). In particular, Lee et al. described the increase risk of hip fractures in PPIs users/BP non-users in comparison with PPIs/BP non-users in a large cohort of Korean subjects (OR: 1.34, 95% CI 1.24–1.44) (65). This was also confirmed by a meta-analysis and quantifying the magnitude of the association between PPIs and the risk of hip fracture. Patients with PPIs had a greater risk of hip fracture than those without PPI therapy (RR 1.20, 95% CI 1.14–1.28, *p* < 0.0001), with a positive relationship between hip fracture risk and doses of PPI taken (66). A recent meta-analysis has explained, the possible mechanisms of fractures induced by PPIs including hypersecretion of histamine and hyperparathyroidism due to hypergastrinemia, as well as mineral and vitamin B malabsorption due to hypochlorhydria (67). Therefore, physicians and patients themselves should be cautious of a higher risk of hip fracture of long-term PPI treatment, it should be encouraged to consider alternative treatment strategies, especially when PPI efficacy is suboptimal.

#### 3.2.4. Mechanical load and bone geometric parameters

The risk of hip fracture is largely dependent on the condition of the bones, with bone structure playing the most important role. Extensive studies have investigated the influence of hip geometric parameters on the risk of hip fracture, especially focusing on the length of the hip axis (HAL), followed by the geometric parameters of the proximal femur, including the femoral neck axial length (FNAL), neck shaft angle (NSA), central edge angle (CEA), femoral neck width, femoral shaft diameter, cortical thickness, cross-sectional area, and cross-sectional modulus. Differences in proximal femur geometry leads to different types of hip fracture. Femoral neck fracture is related to a larger NSA, larger femoral shaft diameter (FSD), larger pelvic outer diameter (POD), longer HAL, longer FNAL, greater neck length-width ratio (NLWR), and larger acetabular width (AW) (68). Recent research based on these findings has shown that compared with patients with femoral neck fracture, patients with intertrochanteric fracture often have a larger CEA because they have longer osteophytes and narrower joint spaces. This makes the impact point between the posterior edge of the acetabulum and the posterior edge of the femoral neck closer to the intertrochanteric region of the femur when they fall (69).

Early mechanical loads also affect the hip joint. The age at which an individual starts walking in infancy may change the shape of their hips, and some characteristics of babies who start walking earlier may reduce the risk of hip fracture. This may be because infancy is the key stage of hip development, and the bone load caused by early walking makes the femoral head and neck larger, the neck shaft angle smaller, and may result in the optimal hip joint configuration (70). Conversely, mechanical unloading decreases bone volume and strength. For instance, being excessively sedentary may negatively affect bone health by disrupting the bone formation-resorption balance, as occurs in immobilized individuals (71), a study performed in 11- to 13-year old boys have shown that changes in sedentary time were negatively related to changes in whole-body bone mineral density (BMD), lumbar spine bone mineral content (BMC), lumbar spine bone area (BA), femoral neck (FN) BMD, and FN BMC (*r* > −0.157; *p* < 0.05) (72). In addition, a cohort study showed that the cortical thickness of 60-year-old women decreased by 6.4% (SD1.1) per decade (*p* < 0.0001), due to atrophic thinning caused by underloading of the upper lateral cortex. Therefore, it is very necessary to do more targeted exercise for people of different ages in order to strengthen fragile bones (8).

Genetic factors (height) play an important role in bone development (33), as height is positively associated with an increased risk of hip fracture (73). In contrast, a recent study showed that a greater amount of age-related height loss is associated with a higher risk of hip fracture in men (74). On the other side, genetic variability affects the skeletal response to unloading, an animal study with different strains of mice proved magnitude of bone loss from immobilization is heritable, and bone transcriptomic response to immobilization is influenced by genetic variation. Thus, genetic factors not only play a major role in the accrual and maintenance of bone mass, but also influence the skeletal response to mechanical unloading. Future studies are suggested to identify genes that are responsible for unloading-induced bone changes thereby to uncovering novel genes responsible for protecting bone from unloading.

#### 3.2.5. Neuromuscular function

A cohort study showed that bone microstructure changes are affected by neuromuscular function. In men, poor physical fitness and lower limb relative appendicular lean mass accelerate cortical bone loss and increase the risk of falls, leading to hip fracture (75). In addition, recent research suggests that people with a fall phobia are more likely to fall in the first 12 weeks after a hip fracture because of their high degree of neuroticism (76). A cross-sectional study reported that the combination of sarcopenia and osteoporosis potentially increases the risk of hip fracture (77). Another established fall factor that should be corrected is a decline in muscle quality in certain areas. The fall risk is related to the mass of the psoas major muscle and the extensor muscle of the spine, and weak psoas and spine extensors may increase the risk of fracture (78). In addition, a cohort study involving 7,575 women aged 75 or older showed that neuromuscular injury (gait speed and tandem walk) and poor vision were important and independent predictors of hip fracture risk (9).

#### 3.2.6. Social and cultural

Though ethnic differences have a well-known effect on the risk of hip fracture, the underlying mechanism has not been identified. One potential factor affecting the ethnic differences in hip fracture risk is interracial differences in social and cultural concepts. People in Asia have a conservative way of thinking, which limits their opportunities to participate in certain activities (79). Similarly, compared with white, low fall rates evident in Chinese cohorts result from greater levels of concern about falling. These findings suggest that the interracial difference in hip fracture incidence may be caused by the different frequency of falls (80). However, a recent study focused on the race-specific association of social environmental factors with hip fracture incidence proved that a greater social stress is associated with a greater hip fracture incidence, with a lower hazard ratio (HR) value in white women [HR 1.04, 95% confidence interval (CI) 1.01–1.08] than Asian women (HR 1.37, 95% CI 1.01–1.86) and native American women (HR 1.84, 95% CI 1.10–3.10) (81). A cohort study from the United States based on data from 11,020 postmenopausal women confirmed this result. After adjustment for confounders, each point higher in social strain was associated with 0.082% greater loss of femoral neck BMD, 0.108% greater loss of total hip BMD and 0.069% greater loss of lumbar spine BMD (*p* < 0.05) (82).

### 3.3. Well-accepted protective factors

Previous studies have confirmed that the protective factors negatively correlated with the risk of hip fracture include hormone replacement therapy, the use of thiazide diuretics, consumption of tea, weight-bearing exercises of relatively low intensity, consumption of food rich in protein, increased calcium and vitamin D intake, and the use of hip protectors (10, 12, 83, 84) (Figure 1).

### 3.4. Recently confirmed protective factors

Diet, nutrition, and physical activity are inextricably linked to hip fracture. Recent studies confirm that the risk of hip fracture can be reduced by the intake of essential nutrients (vitamin C, vitamin K, magnesium, potassium, and unsaturated fatty acids) (Figure 2).

#### 3.4.1. Vitamins

A meta-analysis showed that dietary vitamin C intake reduces the risk of hip fracture, with every 50 mg increase in daily vitamin C intake reducing the risk of hip fracture by 5% (85). According to another meta-analysis, increasing the vitamin C intake reduces the risk of osteoporosis by 33% and increases the BMD of the hip (86), which is further confirmed in a latest study that dietary vitamin C-targeted food intake is negatively associated with the risk of fracture and a decrease in BMD (87).

Vitamin K causes blood coagulation and may greatly affect bone metabolism. The effect of vitamin K on the incidence of hip fracture is controversial. Previous studies have shown that dietary vitamin K intake does not affect the risk of hip fracture (88, 89), However, other studies have shown that every 50-mg increase in vitamin K in the diet reduces the risk of fracture by 3%. Overall, a higher dietary intake of vitamin K reduced the risk of fracture by 22%, and suggest that the protective effect of vitamin K on bone health depend on its effect on bone transport and BMD (90).

#### 3.4.2. Magnesium and potassium

Most epidemiological studies have shown that magnesium intake is associated with higher BMD, although the appropriate doses of calcium and magnesium remain unclarified (91). A meta-analysis has shown that a high-dose magnesium intake does not affect the risk of hip fracture, but that there is a slight correlation between magnesium intake and hip BMD (pooled *r* = 0.14) (92). In addition, a cross-sectional study of 4,000 people in Europe showed that a low dietary intake of magnesium and potassium reduces the BMD of the whole hip (93). A recent South Korean study found that higher levels of potassium in food (3,600–3,800 mg per day) are good for the heart and bones of older women (94). Alkaline potassium salts are thought to prevent the pH homeostasis of bone resorption. Therefore, dietary potassium may prevent bone loss by affecting the acid-base mechanism (95). There is no doubt that nutritional elements are important for bone health protection. While little is known about the influence of trace elements on hip fracture, a study proposed that many trace elements affect bone metabolism. Zinc and copper affect bone turnover and increase bone strength, while magnesium, iron, manganese, boron, and fluoride protect bones. However, excessive intake of protective elements (zinc, fluorine, magnesium, and iron) can also have an adverse effect on bones (96).

#### 3.4.3. Unsaturated fatty acids

A prospective cohort study suggested that low fish intake may increase the risk of hip fracture, and that this association is stronger in men (HR 1.84, 95% CI 1.10–3.08). Fish are rich in nutrients, including high-quality protein, large amounts of unsaturated fatty acids, and phospholipids. Among these nutrients, unsaturated fatty acids play a protective role in hip fracture (97). Although previous studies reported differences in the associations between unsaturated fatty acids and hip fracture, a recent review determined the protective effects of unsaturated fatty acids based on more than a dozen dose-response relationships and showed that dietary n-3PUFA intake is negatively correlated with fracture risk (98). Furthermore, saturated fatty acids are associated with a lower risk of cognitive impairment (99). This effect may be indirectly associated with reduced risks of falls and hip fracture.

### 3.5. Controversial factors

Some factors with no consistent results with respect to the association between them and hip fracture risk, are considered as controversial factors.

### 3.6. Thyroid function

Some recent studies on thyroid function contradict the previous conclusions. Most studies have found that in normal subjects with thyroid function, a decrease in the levels of thyroid stimulating hormone and an increase in the level of free thyroxine are associated with an increased risk of hip fracture (100). In contrast, Siru et al. reported that thyroid stimulating hormone and free thyroxine levels are not associated with incident hip fracture. However, their data supports the hypothesis that older men are less susceptible to elevated levels of thyroid hormones than older women, and identifies sex differences in the associated fracture risk (101). However, a recent study reported that for both men and women, patients with hip fractures whose plasma TSH levels were higher than the median (1.41 mIU/L) at admission after surgery had an increase in mortality at 30 days after operation (102). Therefore, future studies should consider potential sex differences in thyroid function related to the risk of fracture.

### 3.7. Bone turnover markers

Bone turnover markers (BTMs) are a group of proteins and peptides released during bone remodeling that can be found in serum or urine. It was suggested that higher serum levels of procollagen type I N propeptide (PINP) and C-terminal crosslinking telopeptide of type I (CTX-I) were associated with an increased risk of hip fracture (103, 104). However, in a case-control study, S-CTX, and S-PINP did not differ between groups (105). Another study from Marques et al. (106) do not support the routine use of BTM to assess fracture risk in older men and women. However, these studies only assessed those BTMs at single time point, and given the known variability of BTMs, multiple measurements may drive to more substantial evidence of their associations with bone loss and fracture (107). Furthermore, a study has found that higher levels of BTMs were associated with higher cortical porosity and thinner cortices, which may lead to a higher incidence of fracture (108). The relationship between bone turnover markers and fractures and the underlying mechanism needs to be verified by more standardized studies in the future.

### 3.8. Genetics

The mechanism that affects the risk of hip fracture is complex, part of which is attributed to heredity. Studies have shown that genetic factors are mainly focused on gene polymorphisms that affect BMD, turnover, architecture and size. To date, although researchers have determined that vitamin D receptor (VDR) gene, collagen I-α-1 (COLIA1) gene, and estrogen receptor (ER) gene are associated with the risk of osteoporosis (109). However, it is still controversial whether these genes are associated with BMD. The results of a prospective cohort study showed that VDR CC genotype and COLIA1 TT genotype were associated with increased hip fracture risk in Caucasian women, and this association was independent of BMD and age (110). ER gene are not associated with osteoporotic fractures, bone mass, or bone turnover (111). In addition, Studies show that the hip axis length is affected by both the GDF5 gene (112) and the DKK1 gene (113) and whether both genes will increase the hip fracture risk is further confirmed by future studies. In general, individual studies lack sufficient power to identify genetic associations with fracture that are independent of BMD.

In addition to the above referred genes associated with hip fracture. Recent studies have shown that the ABO blood group system may also have effects on bones. However, the influence of the ABO blood group system on hip fracture remains controversial. A recent study suggested that the ABO blood group affects the risk of proximal femoral fracture, as patients with A blood group are more likely to incur intracapsular fractures than those with other blood types (114). In contrast, Toro et al. found that the ABO blood group system does not influence hip fracture pattern, but that patients with A blood group are more prone to fracture than those with type O blood (115). The mechanism by which the ABO blood group system affects hip fracture is unclear. A study showed that Blood type O was associated with higher BMD (116). Contrarily, a cross-sectional study showed that postmenopausal women in North India who do not have O blood group have a higher BMD than those with type O blood. It has been reported that ABO antigen may exist in bone tissue, suggesting a biological relationship between ABO blood group and fracture (117). In addition, the ABO blood group is related to the incidences of cardiovascular diseases and cancer (118, 119), which are considered risk factors for hip fracture. Therefore, further work is needed to assess the association between ABO blood group and risk of hip fracture, as well the underly mechanism whereby blood group affect fracture risk.

### 3.9. Concrete preventive measures

Based on the above-mentioned influencing factors, we suggest the following intervention measures and discuss the problems involved in optimizing the strategies for preventing bone loss and hip fracture.

#### 3.9.1. Fall prevention

Falls are the main cause of hip fracture in the older adult population. It is estimated that 30% of people older than 65 years fall each year and 87% of fractures are caused by falls; therefore, the prevention of falls is an important means to reduce the incidence of hip fracture (120). The external environmental factors contributing to falls include balustrade stairs, loose carpets, poor lighting, and potholed floors, landing on or near the hip, orientation of the fall (backward or to the side), greater potential energy of the faller, reduced soft-tissue padding over the hip. Individuals should avoid walking in these places or walk carefully. Slowing down also greatly reduces the incidence of falls (121, 122). In recent years, the global climate environment has deteriorated sharply, and the impact of meteorological factors and air pollutants (MFAPs) on hip fracture is becoming increasingly obvious. Different MFAPs (average temperature, day rainfall, wind speed, daily snowfall, and particulate matter 2.5) are positively correlated with the risks of falling and hip fracture. Therefore, it is necessary to focus on the protection of the environment and strengthen the public's cognition of MFAPs to reduce the occurrence of hip fractures (123). It is also important to identify high-risk groups that are prone to falls and have an increased risk of hip fracture, such as older adults, women, patients with a history of falls, lower limb dysfunction, neuromuscular injuries, less physical activity, visual impairment, stroke, neurodegenerative diseases (Parkinson's and Alzheimer's disease), and mental disorders (depression) (124, 125). Patients at high risk of falls should wear a hip protector (126). However, the use of physical restraint increases the incidence of falls and because some people do not have good compliance, the hip protector can't play a full role (11, 127). Therefore, it is necessary to further improve the comfort of protective tools to improve the protection against hip fracture.

Internal mental health is also an issue that requires attention. Patients with a fall phobia are more likely to fall in the first 12 weeks after hip fracture because of their neuroticism (76). Therefore, it is essential to provide all-round psychological counseling to people who have fallen.

#### 3.9.2. Selection and use of medicines

The administration of estrogen, glucocorticoids, aromatase inhibitors, and psychotropic drugs (such as benzodiazepines and barbiturates) is associated with decreased BMD of the hip and an increased risk of falling. In addition, recent studies have found that the choice of anti-osteoporotic drugs is important. Anti-osteoporotic drugs are generally divided into three categories: calcium supplements, bone resorption inhibitors, and bone-forming agents. Bisphosphonates [alendronate (ALN), risedronate, ibandronate, etidronate and zoledronic acid], non-bisphosphonates [denosumab (DEN) and raloxifene], and bone-forming agents (teriparatide and romosozumab) have protective effects against hip fracture (128). However, questions have recently been raised about the efficacy of bisphosphonates in preventing hip fracture, as frail older adults who use bisphosphonates still reportedly develop fractures (129).

Numerous recent studies show that non-bisphosphonates have a more obvious protective effect than bisphosphonates. Bisphosphonates mainly work in women, especially postmenopausal women, and the protective effects of bisphosphonates on hips vary with the type of drugs. ALN may be more effective in the secondary prevention of hip fracture (130). Compared with ALN alone, romosozumab followed by ALN was associated with lower incidences of hip fractures among East Asian patients (131). The Postmenopausal women can safely transition from a bisphosphonate to DEN, which is more effective at improving BMD than continuing with a bisphosphonate (132). DEN is more effective than bisphosphonates in the treatment of total hip BMD in patients with glucocorticoid-induced osteoporosis (133, 134). Teriparatide is more effective than risedronate in increasing the BMD of the femoral neck and total hip (135). Furthermore, long-term bisphosphonate treatment should be converted to teriparatide in high-risk patients with hip fracture (136).

In summary, the selection and use of medicines have important implications regarding hip fracture, and high-risk groups should use drugs that have a protective effect against hip fracture; such drugs include thiazide diuretics (hydrochlorothiazide, bendroflumethiazide, or fixed drug combinations containing a thiazide) and sequentially bisphosphonates and non-bisphosphonates s treatment for osteoporosis should be suggested.

#### 3.9.3. Formulation of the dietary treasure book of professionals

A moderate dietary intake of vitamin C, vitamin K, magnesium, and potassium is good for hip bone health, while an excessive intake of protective elements (vitamin D, zinc, fluorine, magnesium, and iron) has an adverse effect on bone health. Therefore, the control of nutrient intake is very important. Interestingly, nutrients play an important role in dental health, and a decrease in the number of natural teeth is associated with low BMD and hip fracture, with a relative risk of hip fracture of 12% (137). In addition, a retrospective cohort study showed that eating highly nutritious foods (fruits, vegetables, and whole grains) reduces the risk of traumatic fracture, while eating high-energy foods (soft drinks, potato chips, French fries, meat, and desserts) and a high total energy intake are not associated with fracture (138). However, specific studies on how dietary patterns affect hip bone health are very limited; future studies should develop a dietary guide for people at high risk of hip fracture.

Low body weight is an important factor in hip fracture, and it is well known that low body weight may be caused by malnutrition, intentional weight loss, poor appetite, and small body size. Most studies have focused on the simple measurement of body weight, while few studies have evaluated lean body weight or fat content. One study showed that fat content in patients with gestational diabetes mellitus is related to bone health, but not to body mass index (139). Therefore, future research on fat content is very important to reduce hip fractures. For underweight people, poor appetite, lack of nutrition, and intention to lose weight can all be subjectively corrected to achieve a normal weight and promote health. The latest data show that weight gain is a protective factor for hip fracture (140). However, excessive weight gain may lead to abdominal obesity and general obesity, which increase the risk of hip fracture (141). In addition, in the middle-aged and older adult population, a larger hip circumference has a protective effect against hip fracture, which may be related to better muscle strength and internal factors affecting bone strength (142). Therefore, further studies are needed to identify easily measurable factors influencing hip fracture.

#### 3.9.4. Reasonable adjustment of exercise intensity and lighting time

Appropriate physical activity effectively helps older adults improve their physical function and reduce the risk of fall-related injuries. Among the mechanisms related to hip fracture, many studies have emphasized the importance of physical activity. A case-control study showed that quality of life is closely related to hip fracture. Furthermore, long-term exercise and physical intervention can improve physical fitness (143). However, the prevention of hip fracture by exercise has not been confirmed. A randomized controlled trial showed that exercise self-intervention had no significant effect on action-related disability and falls (144). However, another study showed that walking for an hour a day and participating in yoga and physical activity reduced the risk of hip fracture by 15–20% (145). Regular Tai Chi Chuan would appear to be of benefit (146). Outdoor sports increase the time spent in the sun, which is good for bone health, but prolonged exposure to sunlight can lead to photochemical damage (147). In addition, a combination of moderate and intense physical activity may improve bone mass and reduce the risk of hip fracture (148, 149). A cohort study found that farmers who engaged in higher levels of physical activity are less likely to incur hip fractures (150). In addition to the amount of activity, changes in regular physical activity status are associated with the risk of hip fracture; those who became active and those who were always active exhibited a 0.24/1,000 person-years reduction in incidence rate and a 0.39/1,000 person-years reduction in incidence rate, respectively (151). Therefore, regular physical activity at an appropriate intensity is beneficial for skeletal health and is a preventive factor for hip fracture.

## 4. Discussion

This review summarizes the recently identified risk factors that are positively correlated with the risk of hip fracture, including AN, hyponatremia, hypokalemia, loop diuretics, analgesics, high doses of vitamin B and vitamin D supplements, Proton pump inhibitors, early mechanical loads, larger NSA, CEA, FSD, POD, longer HAL, FNAL, greater NLWR and AW, neuromuscular damage, climate deterioration, adventure way of thinking, and high social stress.

Newly identified protective factors associated with a reduction in the risk of hip fracture include a moderate dietary intake of vitamin C, vitamin K, magnesium, potassium, and unsaturated fatty acids, dental health, large hip circumference, and moderate or high-intensity physical exercise (yoga, Tai Chi Chuan) and regular physical activity, sequentially using of bisphosphonates and non-bisphosphonates, the use of thiazide diuretics. In addition, there has been recent controversy about the associations of thyroid function, bone turnover markers and genetics with hip fractures, which needs to be verified by more standardized studies.

In summary, maintaining the normal physiological structure of hip bones, controlling infant walking time, improve the exercise time of the elderly, monitoring height, neuromuscular quality are key measures to prevent or delay the onset of hip fracture, while the use of antihypertensive drugs with different sites of action, analgesics of different strengths, PPIs and psychotropic drugs in clinical practice should be given reasonable doses according to pharmacokinetics and overall individual metabolism, and closely monitored for drug side effects to meet the safety standards of these drugs for hip patients.

In addition, in daily life and physical examination, attention should be paid to the effects of trace elements, unsaturated fatty acid intake and related ion concentration on bone health. At the same time, with the improvement of economic level and health awareness, the elderly and teenagers often take certain vitamins to maintain the balance of energy metabolism. However, due to the lack of safety awareness and scientific advice, people recognize that non-toxic, non-dose and unlimited “health products”, such as vitamin supplements, may cause some diseases after long-term use. Therefore, vitamin intake and supplement should be based on their own needs and scientific advice to prevent the occurrence of fractures.

## 5. Conclusion

Hip fractures constitute one of the most important causes of morbidity and mortality among the elderly, and are associated with considerable and increasing economic burden. Given the increasing per capita life expectancy and the progressive increase in the tendency to aging, it is foreseeable that hip fractures will be one of the major challenges facing the global public health in the coming years.

This review provided a systematic description of the influencing factors and preventive measures of hip fracture, which summarizes the recently identified diseases related to the risk of hip fracture, including disease, medication, mechanical load, neuromuscular mass, ions related to energy metabolism, abnormalities of vitamins and unsaturated fatty acids, and the influence of social background.

Future research should optimize intervention strategies by improving the comfort of protective tools and providing guidance on psychological counseling after falls, appropriate selection of therapeutic drugs and appropriate doses of therapeutic drugs and more targeted and reasonable exercise for people of different ages to optimize the intervention strategy. It is also important to further develop reasonable dietary guidelines and to strengthen the identification of easily measurable risk factors for hip fractures.

## Author contributions

YY and YW drafted the manuscript. XH assisted in the writing. FT edited the manuscript to its final version. All authors read and approved the final manuscript.
