# Supplementary material for: Recent advances in the identification of related factors and preventive strategies of hip fracture
Source: Front Public Health. 2023 Mar 13;11:1006527. doi: 10.3389/fpubh.2023.1006527 (PMC10040558; doi:10.3389/fpubh.2023.1006527)
Supplement: Supplementary file 1 [file Table_1.pdf]

Appendix. Comprehensive search strategy for representative databases

**Search terms**	
**PubMed search**	
1	"hip fractures"[MeSH Terms] OR ("hip"[Title/Abstract] AND "fractures"[Title/Abstract]) OR "hip fractures"[Title/Abstract] OR ("hip"[Title/Abstract] AND "fracture"[Title/Abstract]) OR "hip fracture"[Title/Abstract]
2	("risk factors"[MeSH Terms] OR ("risk"[All Fields] AND "factors"[All Fields]) OR "risk factors"[All Fields] OR ("risk"[All Fields] AND "factor"[All Fields]) OR "risk factor"[All Fields] OR ("influence"[All Fields] OR "influenced"[All Fields] OR "influences"[All Fields] OR "influencing"[All Fields]) OR "correlate*"[All Fields])
3	1 AND 2
**Cochrane library search**	
1	"hip fractures" OR CVD OR "hip cataclasis" OR "hip ossium" OR stroke OR "pygal fractures"
Filter: tiab, keyword	
2	"risk factor?" OR "influenc* factor?" OR "correlate*"
Filter: tiab, keyword	
3	1 AND 2
**Web of Science search**	
1	"hip fractures" OR CVD OR "hip cataclasis" OR "hip ossium" OR stroke OR "pygal fractures"
Filter: None, Topic	
2	"risk factor" OR "influencing factor" OR "correlate*"
Filter: None, Topic	
3	1 AND 2
